# The effect of parental regulatory focus on the loneliness stigma of college children

**DOI:** 10.1186/s12889-024-17714-8

**Published:** 2024-01-23

**Authors:** Zhiguang Fan, Xiaoli Shi, Wei Zhang, Bin Zhang

**Affiliations:** 1 Department of Psychology, Shaoxing University, Zhejiang, China; 2 School of Education, Jilin International Studies University, Jilin, China; 3 Higher Education Press, Beijing, China; 4 School of Marxism, Changchun University of Chinese Medicine, Changchun, China

**Keywords:** Promotion focus, Prevention focus, Loneliness stigma, Intergenerational transmission, APIM

## Abstract

**Background:**

The present study aimed to examine the relationship between regulatory focus and loneliness stigma, as well as the intergenerational transmission of the two. Specifically, the study analyzed the effects of fathers’ and mothers’ regulatory focus on their own and their spouses’ stigma of loneliness. In addition, a mediation model was constructed to explore how parents’ regulatory focus influences their children’s stigma of loneliness and the potential mediating mechanisms involved.

**Methods:**

Questionnaires were distributed to 470 college students and their parents, employing the Regulatory Focus Questionnaire (RFQ) and the Stigma of Loneliness Scale (SLS) to collect data.

**Results:**

The analysis of intergenerational transmission effects revealed that parents’ regulatory focus and loneliness stigma significantly and positively predicted children’s regulatory focus and loneliness stigma, respectively. The Actor-Partner Interdependence Model (APIM) elucidated that both fathers’ and mothers’ promotion focus exerted significant influence on both actor and partner’s loneliness stigma. Furthermore, the mediation model analysis indicated that parents’ loneliness stigma, along with children’s regulatory focus operate as mediators in the influence of parental regulatory focus on loneliness stigma of their college-aged offspring.

**Conclusions:**

From a familial context, this study, investigated the association between regulatory focus and loneliness stigma, along with the mediating roles within parent-child groups and couples. The findings enhanced our comprehension of the interrelation between regulatory focus and loneliness stigma, underpinned by empirical evidence.

## Background

Loneliness is a distressing psychological experience that arises when an individual perceives a discrepancy between expected and actual social relationships, leading to dissatisfaction with their sense of belonging [1]. While the impact of loneliness on individuals is multifaceted, encompassing potential negative and positive aspects [2], it is predominantly perceived as a precursor to deteriorating health and various maladaptive behaviors [3]. This view, coupled with its association with negative affective states and adverse social experiences, has triggered people’s denial, rejection, and stigmatization toward loneliness. People with high levels of loneliness are often underestimated in their achievements, ability to connect with others, social skills, and interpersonal appeal in contrast to their non-lonely counterparts. They are often stigmatized with negative labels such as unpopular, weak, inauthentic, and unsociable [4].

Loneliness stigma encompasses the negative stereotypes, prejudices, and discrimination that individuals possess toward loneliness and being socially inferior due to loneliness [5]. The stigma of loneliness manifests in two distinct forms: public and self-directed. Stigmatic beliefs associate loneliness with attributes such as weakness, helplessness, and shame [6]. Public stigma can result in devaluation, discrimination, and rejection of the lonely people, thus potentially exacerbating their isolation [7]. Self-stigma, involving personal endorsement and integration of these negative beliefs, can amplify the adverse consequences of loneliness and inhibit the propensity to seek help [8]. Previous research has revealed that an individual’s fear and worry about negative evaluations associated with loneliness can intensify loneliness and discourage prosocial behavior [9].

### The relationship between regulatory focus and loneliness stigma

Previous studies have delved into the root causes of stigma from the perspectives of culture, policy, individual factors, and situational factors [10, 11]. With these, motivational factors have attracted increasing attention among scholars [12]. Regulatory focus theory, as a leading framework in the field of motivation, has been widely applied to studies on interpersonal relationships and stigma [13]. This theory assumes that individuals self-regulate their cognition and behavior to attain desired goals, which are channeled through two primary motivational systems: promotion focus and prevention focus [14]. Promotion focus involves regulation geared towards advancement, emphasizing ideals and ambitions, and focusing on potential gains during goal attainment. In contrast, prevention focus centers on the need for security, prioritizing responsibility, safety, and avoidance of potential losses.

Regulatory focus theory suggests that different regulatory focuses lead to distinct psychological outcomes, affecting perception of loneliness stigma in various ways. Promotion-focused individuals tend to be attuned to positive results, strive for high-level goals, and experience emotions on a spectrum from cheerfulness to dejection. Conversely, prevention-focused individuals are more susceptible to negative results, prefer to take a cautious stance in goal pursuit and are prone to emotions ranging from tranquility to agitation [15]. Therefore, regulatory focus acts as an information “filter”, selectively shaping the intake and processing of external stimuli [16]. Specifically, Promotion-focused individuals are inclined to employ proactive language to describe loneliness and construct more positive self-frames, thereby helping to diminish loneliness stigma [17]. Prevention-focused individuals, on the other hand, are likely to concentrate on the adverse implications of loneliness and its perceived threats, potentially intensifying stigmatization [18].

Moreover, individuals with different predominant regulatory focus also vary in their responses to unmet belongingness needs. Promotion-focused people proactively seek opportunities to reforge social bonds in the face of social rejection, whereas prevention-focused counterparts exhibit greater caution, tend to withdraw, and avoid social interaction [19]. Consequently, regulatory focus has the potential to influence experiences of loneliness which is one predictor of loneliness stigma [5]. In Park et al.'s study, it was observed that experiences of social exclusion can trigger a shift from a promotion focus to a prevention focus. In this process, individuals experiencing high levels of loneliness tend to develop a stronger prevention motivation and a diminished promotion motivation [20]. Consequently, promotion focus is hypothesized to be inversely related to loneliness stigma, whereas prevention focus may be positively associated with it.

### The intergenerational transmission of regulatory focus

As shown in regulatory focus theory, parenting styles and parent-child relationship patterns are pivotal in shaping the regulatory focus tendencies of the children [21]. Evidence from cross-sectional and longitudinal studies indicates that parents’ regulatory focus can influence their own parenting choices, which in turn affects the development of their children’s regulatory focus [22, 23]. Promotion-focused parents favor a bolstering mode that not only facilitates the child in gaining pleasure and favorable outcomes, but also benefits him or her in focusing on progress and growth and in gaining achievement, hope, and ambition [24]. Conversely, prevention-focused parents are prone to adopt a prudent mode or critical/punitive mode and often resort to harshly critical and punitive parenting styles, resulting in children who may be more concerned with protection, safety, and responsibility [21]. Furthermore, Andre et al.'s study found that adolescents’ perceptions of their parents’ regulatory focus can influence the development of their regulatory focus [25]. This suggests a probable intergenerational transmission pathway for regulatory focus, where the orientation of parents directly influences and potentially molds that of their children. Biogenetic factors may also contribute to the intergenerational transmission of regulatory focus. There is a neural and physiological basis for the formation of regulatory focus [26]. For example, it has been observed that individuals with high promotion focus exhibits lower levels of ventral striatum response to gain cues, whereas prevention focus did not correlate with ventral striatum response to gain cues [27]. Furthermore, a promotion focus is associated with greater left frontal cortex activity, whereas a prevention focus is related to right frontal cortex activity [28]. Neurophysiological studies have documented that parents and their children exhibit analogous patterns in functional and structural brain networks [29], indicating that specific neural mechanisms may play a role in passing regulatory focus traits down from one generation to the next.

### Intergenerational transmission of loneliness stigma and mediation modeling

At present, direct indication of the intergenerational transmission of loneliness stigma is currently lacking. However, relevant research highlights that parents can transmit their loneliness stigma to their children and exert a long-term effect on them through multiple channels such as social learning, family education, parent-child interaction, and hereditary factors [30]. On the one hand, children can acquire attitudes similar to those of their parents through observational learning or parental education [31]. Parents with high levels of loneliness are squint towards negative parenting style, which has a detrimental effect on the development of their children’s communication skills and social competence [32]. This can leave children more prone to social isolation, exacerbating their sense of loneliness and subsequently increasing their susceptibility to loneliness stigma [33].

On the other hand, the intergenerational transmission effect of loneliness has been verified in studies on various age groups [34–36]. Several empirical studies revealed that parents’ loneliness can impact both their own and their children’s psychology and behavior profile [32]. Loneliness stigma, defined the social aspect of loneliness, is inherently linked to the phenomenon. Typically, individuals with high loneliness tend to encounter higher loneliness stigma [37]. Consequently, in transmitting their own sense of loneliness to their offspring, parents may also inadvertently transfer the accompanying stigma. Additionally, studies suggest a neurophysiological foundation for stigma comparable to that of regulatory focus [38], inferring that genetic factors might similarly contribute to the intergenerational perpetuation of loneliness stigma.

### APIM model for the impact of regulatory focus on loneliness stigmatization

Although studies have examined the relationship between regulatory focus and loneliness at the individual level [39], no study has examined the effect of regulatory focus on spouses’ loneliness, particularly loneliness stigma. Furthermore, the conjugal bond is considered to be one of the most typical dyadic relationship which characterizes the importance of interdependence. The two parties in a dyadic relationship are non-independent and interact with each other [40]. Given the close association and frequent interactive communication between couples, there is significant potential for mutual influence on each other’s thoughts, feelings, and behaviors as the relationship develops [41]. Consequently, it is plausible to propose that the loneliness stigma inherent in one spouse may be impacted by the other spouse’s regulatory focus.

The most commonly employed methodology for analyzing actor-partner effects in pairwise relationships is the actor-partner interdependence model (APIM) [42]. The APIM has seen widespread application in the study of couple relationships. For example, Rousseau et al. revealed that both actor and partner effects of the mother’s regulatory focus held for the Helicopter parenting style; however, for fathers, only the partner effect of the prevention focus was significant [43]. In a study by Segel-Karpas et al., it was discovered that cynical hostility can affect the loneliness of individuals and their spouses [44]. Consequently, drawing on the findings of prior APIM-driven studies on regulatory focus and interpersonal relationships, it is postulated that the partner effects of both fathers’ and mothers’ regulatory focus are indeed present and influential.

### Present study

The current study aimed to explore the effect of parental regulatory focus and the associated stigma of loneliness on their college-aged children within the context of intergenerational transmission. College students tend to place significant emphasis on interpersonal relationship and feelings of loneliness in a collectivist environment [45], potencially giving rise to distinct manifestations of loneliness stigma that differ from those observed in other adult populations. Kerr et al.'s research indicated a marked presence of stigma towards lonely individuals among college students, whereas less pronounced evidence of such stigmatization was noted in more heterogeneous U.S. adult samples [37]. Therefore, this study focused on college students and their parents as the research cohort, examining the parental influence on their progeny’s perceptions. Based on the theory of regulatory focus and the related research results of intergenerational transmission, we proposed the following hypotheses:

#### Hypothesis H1

The promotion focus is inversely related to stigma of loneliness;

#### Hypothesis H2

The prevention focus positively predicts stigma of loneliness;

#### Hypothesis H3

Regulatory focus shows intergenerational transmission effect;

#### Hypothesis H4

The stigma of loneliness is subject to intergenerational transmission effect;

#### Hypothesis H5

**Fig. 1:**
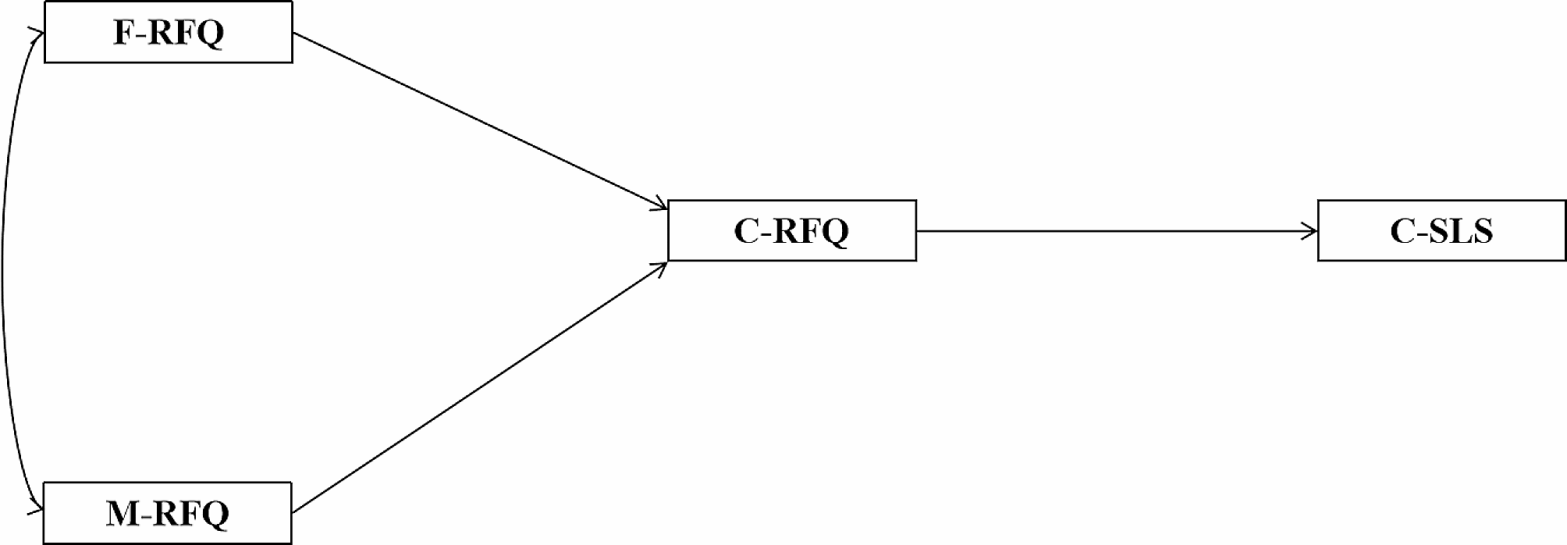
A mediation model of the intergenerational transmission of RFQ and its impact on SLS in college children **Note**: F, Father; M, Mother; C, Children; RFQ, Regulatory Focus Questionnaire; SLS, Stigma of Loneliness Scale

**Fig. 2:**
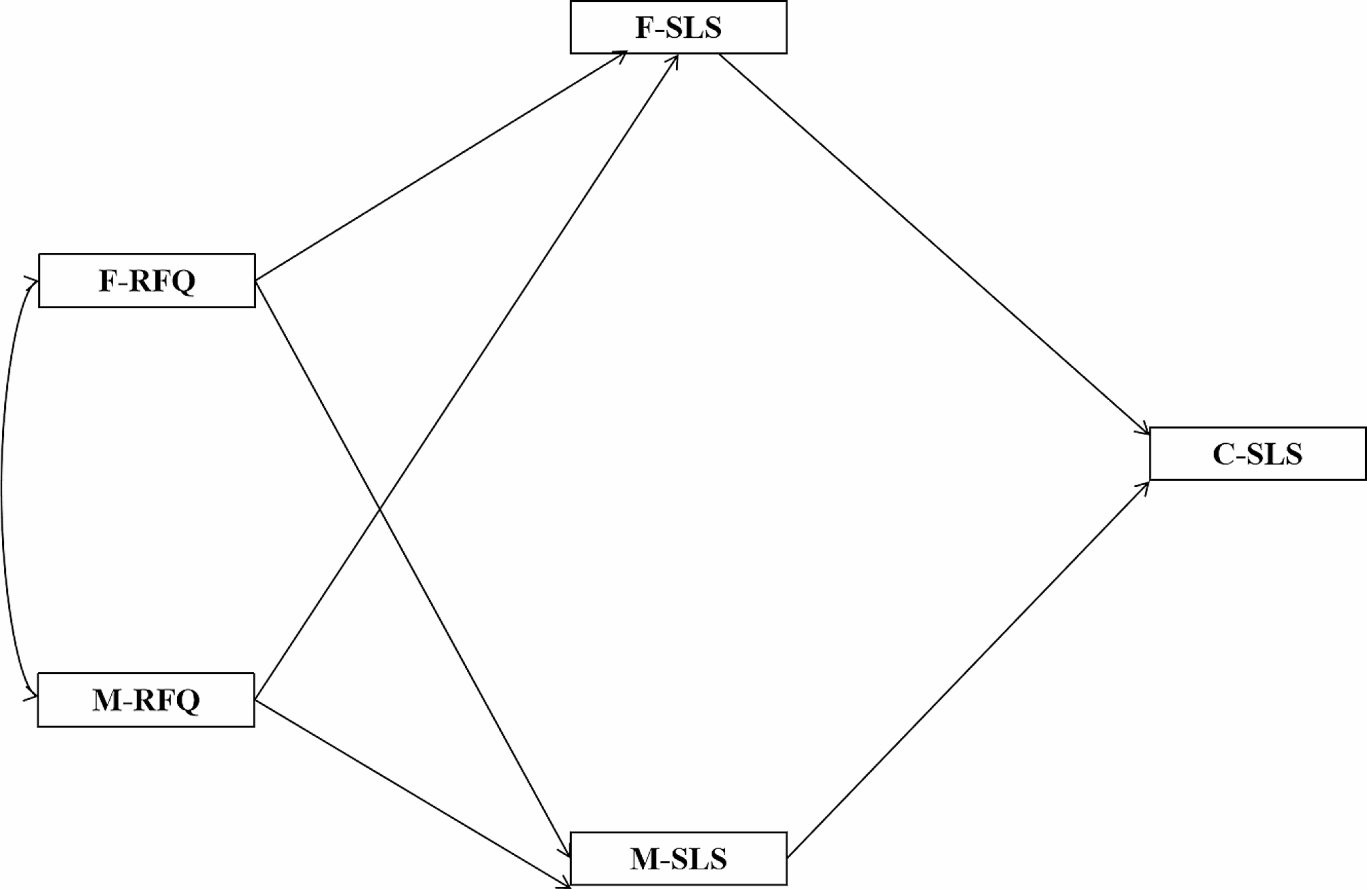
A mediation model of the intergenerational transmission of SLS and the effect of parents’ RFQ on their SLS **Note**: F, Father; M, Mother; C, Children; RFQ, Regulatory Focus Questionnaire; SLS, Stigma of Loneliness Scale

The father’s or mother’s regulatory focus can affect not only their loneliness stigma (actor effect) but also their spouse’s loneliness stigma (partner effect);

Furthermore, according to the family systems theory, elements in a family are mutually influenced, which in turn makes interconnections between couple and parent-child subsystem [46]. Therefore, parental regular focus can shape their children’s regulatory focus and loneliness stigma as similarly the regular focus of children of college students affects their own stigma of loneliness. Given this framework, the present study synthesizes hypotheses H1, H2 and H3 to propose that parental regular focus can affect children’s regular focus through intergenerational transmission, and then indirectly impact children’s loneliness stigma (see Fig. 1). In conjunction with hypotheses H1, H2, H4 and H5, it is speculated that parents’ regular focus can affect their own and their spouses’ loneliness stigma, and then children’s loneliness stigma through the intergenerational transmission of loneliness stigma (see Fig. 2). Accordingly, a mediating model has been formulated to explore how parental regulatory focus impacts children’s stigma of loneliness. Building on this model, the following additional hypotheses are asserted:

#### Hypothesis H6

Children’s regulatory focus mediates the relationship between parental regulatory focus and the children’s loneliness stigma (see Fig. 1);

#### Hypothesis H7

Parents’ stigma of loneliness mediates the relationship between parental regulatory focus and the child’ s stigma of loneliness (see Fig. 2). 

#### Hypothesis H8

**Fig. 3:**
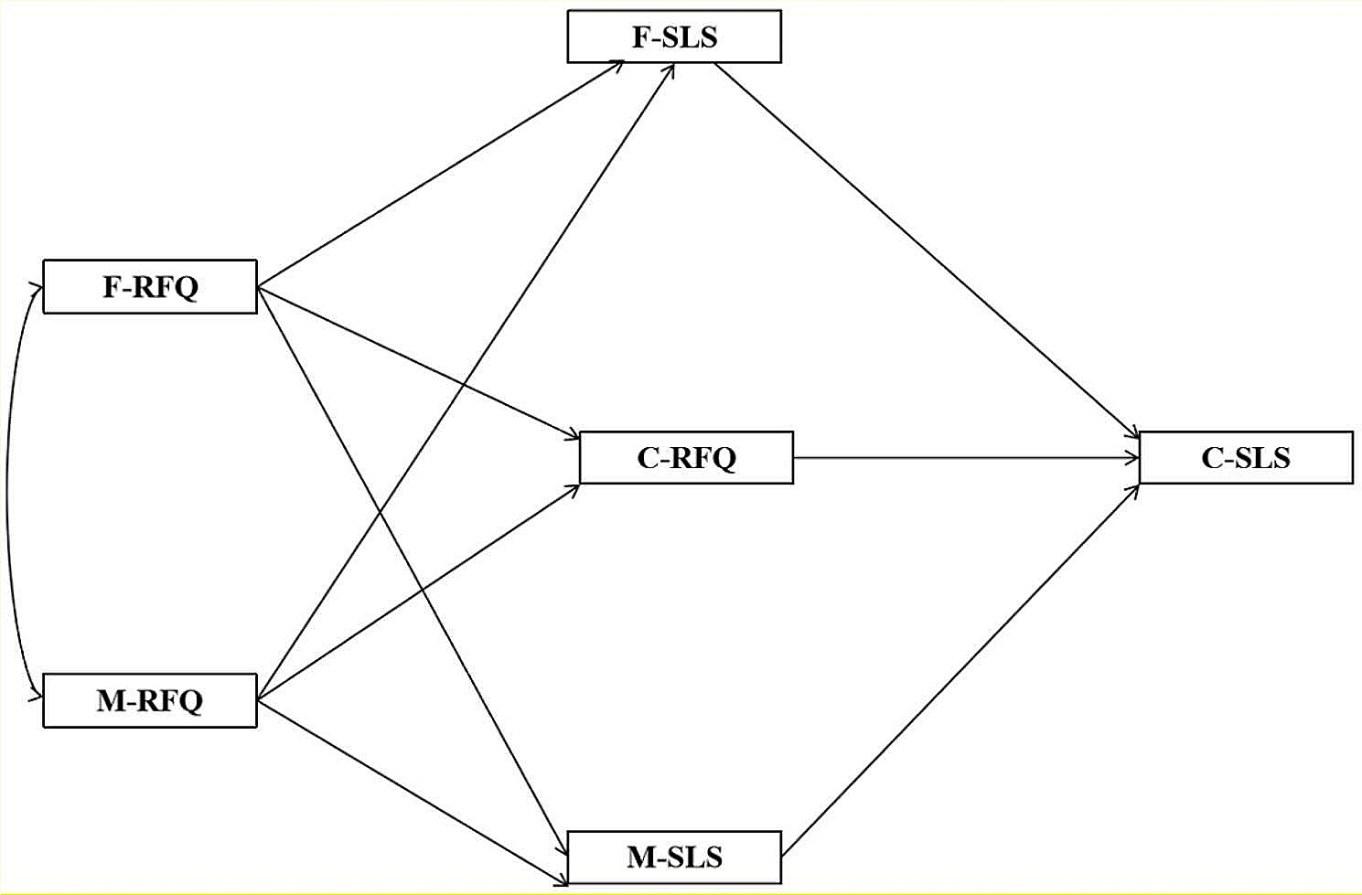
The effect of parental RFQ on SLS in college children and the mediating role of parental SLS and children’s RFQ **Note**: F, Father; M, Mother; C, Children; RFQ, Regulatory Focus Questionnaire; SLS, Stigma of Loneliness Scale

Both parents’ stigma of loneliness and the child’s regulatory focus jointly mediate the relationship between parental regulatory focus and the child’s stigma of loneliness (see Fig. 3).

To examine the above hypotheses, the study employed the Regulatory Focus Questionnaire (RFQ) and Stigma of Loneliness Scale (SLS) to collect data from college students and their parents. In addition to assessing the direct relationship between regulatory focus and loneliness stigma, this investigation also analyzed the dynamics at the individual, parent-child, and couple levels to better understand the mediating mechanisms at play. The findings are expected to offer a theoretical basis for the development of targeted intervention strategies. To further explore the influence of regulatory focus on children’s loneliness stigma and its mediation mechanism, this study analyzed the relationship between regulatory focus and loneliness stigma at the individual, parent-child, and couple levels, expecting to provide a theoretical reference for the development of related intervention practices.

## Methods

### Participants

This investigation was conducted at a university in China. The researcher first communicated with 5 counselors at the school, providing them with the proposal, recruitment poster, and informed consent form for this study. With their consent, the conselors disseminated an e-recruitment poster within their WeChat group. The researchers registered the enrolled students individually and assigned family numbers. The study was facilitated through a web-based survey. College students who volunteered to participate in the survey were required to fill out the child version of the questionnaire and send the web survey link of the parent version to their fathers and mothers, who answered the questionnaire separately and independently. Repeated responses from the same IP address were prohibited to prevent substitution. All subjects were required to read and confirm the informed consent form before starting to answer the questionnaire. To ensure the anonymity of the study, all subjects were only asked to fill in their family number. At the end of the survey, the researchers removed invalid data based on the following criteria. (a) incorrect family numbers disrupting the match of data between family members; (b) incomplete family data sets due to a family member not participating; and (c) indications of inattentive responses, such as identical choices for reversed questions, or completion times falling beyond two standard deviations from the mean [47].

A total of 523 university students were recruited for the study and 470 valid household data points were retrieved. Among them, 300 families (63.83%) were from urban areas and 170 families (36.17%) from rural areas. The participants consisted of 201 male (42.77%) and 269 female (57.23%) students, with ages ranging from 17 to 25 years old and a mean age of 20.37 (SD = 2.01 years). For parental education level, 52 fathers had elementary school education or below (11.28%); 137 had completed junior high school (29.15%); 128 had finished high school or vocational schools (27.23%);67 had attended junior college (14.26%); 70 held bachelor’s degree (14.89%); and 15 had postgraduate degree (3.19%). their ages ranged from 40 to 62 years old, with an average age of 47.58 ± 4.53. The mothers’ literacy levels ranged from elementary school and below 70 ( 14.89%), junior high school 154 (32.77%), high school or junior college 117 (24.89%), college 52 (11.06%), undergraduate 69 (14.68%), and postgraduate and above 8 (1.70%); The age is between 38 and 60 years old. The average age is 46.05 ± 4.38.

## Measurements

### Regulatory focus questionnaire (RFQ)

The RFQ is the most commonly used instrument for assessing an individual’s regulate orientation tendency [48]. The Chinese version of the scale has demonstrated robust reliability and validity across diverse populations. The RFQ consists of 11 items, categorized into two sub-scales, the promotion scale and the prevention scale. The promotion scale consists of 6 items and the prevention scale consists of 5 items. The scale is scored on a 5-point scale, with items 1, 2, 4, 6, 8, 9, and 11 being reverse-scored. The sum of the individual items of each sub-scale is the total score of the sub-scale, and higher scores indicate higher levels of promotion focus or prevention focus in individuals. In this study, the Cronbach’s α coefficients for the promotion focus sub-scale were 0.74, 0.71, and 0.73 for the sample of fathers, mothers, and children of college students, respectively and the Cronbach’s α coefficients for the prevention focus sub-scale were 0.74, 0.72, and 0.76 for the three populations, respectively.

### Stigma of loneliness scale (SLS)

The SLS is an established measure for evaluating an individual’s loneliness stigma [5]. The validity of the SLS has been confirmed among college students and middle-aged adults in China. The SLS consists of 10 items categorized into Self-Stigma of Loneliness (SSL) and Public Stigma of Loneliness (PSL) dimensions. Respondents rate each item on a 5-point Likert scale (1 = “strongly disagree”; 5 = “strongly agree”). The sum of the scores for each item is the total score, with higher scores indicating higher levels of public stigma and self-stigma of loneliness by the individual. The Cronbach’s α coefficients for SLS in this study were 0.94, 0.94, and 0.94 for the sample of fathers, mothers, and children of college students, respectively; for SSL, the Cronbach’s α coefficients were 0.91, 0.91, and 0.90 for the three sample populations; and for PSL, the Cronbach’s α coefficients were 0.92, 0.91, and 0.92 for the three sample populations.

### Data analysis

First, the study examined the relationship between regulatory focus and the loneliness stigma of fathers, mothers, and their college children using Pearson correlation analysis. Subsequently, the linear mixed model (LME) was applied to conduct an intergenerational transmission effect analysis. The family was taken as the subject variable, while parents’ regulatory focus and loneliness stigma were taken as independent variables, children’s regulatory focus and loneliness as dependent variables, and children’s gender and residence (urban or rural) as control variables.

Third, the study conducted the APIM analysis in the APIM_SEM online program [49]. Concretely, the actor effect refers to the effect of fathers’ and mothers’ regulatory focus on their own loneliness stigma. The partner effect is the effect of fathers’ and mothers’ regulatory focus on spouses’ loneliness stigma. The children’s gender and family residence were incorporated as control variables. The k-value is the ratio of the partner effect to the actor effect. Only when both the father’s and mother’s standardized actor effect values were greater than 0.10 could the k-value be calculated [50]. If the confidence interval of the k value includes 0, it means that the pairwise pattern is the actor-only pattern, while if includes 1, it is the couple pattern, and if the − 1 is included, it is the contrast pattern [50]. In addition, to examine whether the pairwise relationship was distinguishable, the study included a chi-square analysis after restricting the actor and partner effects of fathers and mothers to be equal. If the *p* value was less than 0.20, the pairwise relationship would be distinguishable [50].

Fourth, to test the mediation model using AMOS 24.0. The mediation model includes the father’s and mother’s regulatory focus as independent variables, the child’s loneliness stigma as the dependent variable, and the father’s and mother’s loneliness stigma and the child’s regulatory focus as mediating variables. The criteria for a good fit of the model are: χ^2^/df<3, RMSEA<0.08, SRMS<0.05, CFI、TLI、IFI、GFI>0.90 [51]. Additionally, the Bootstrap resampling technique was employed, with 5,000 replications, to validate the significance of the indirect effects within the model. Indirect paths were deemed significant if the 95% confidence interval did not include zero, thereby corroborating the mediating relationship.

## Results

### Correlation analysis of regulatory focus and loneliness stigma

Correlational analyses between parents’ and college-aged children’s regulatory focus and loneliness stigma revealed (see Table 1) that promotional focus exhibited a significant positive correlation among fathers, mothers, and their college-going offspring. Similarly, a substantial positive correlation of prevention focus was also observed across these three groups. Furthermore, a significant negative correlation was found between regulatory focus and loneliness stigma among fathers, mothers, and the college students themselves.

**Table 1:** Results of a Correlation Analysis of Parents’ and College Children’s Regulatory Focus with Loneliness Stigma

	1	2	3	4	5	6	7	8	9
1. F-Promotion	-								
2. F-Prevention	0.31^**^	-							
3. F-Stigma	-0.47^**^	-0.39^**^	-						
4. M-Promotion	0.41^**^	0.18^**^	-0.26^**^	-					
5. M-Prevention	0.25^**^	0.40^**^	-0.33^**^	0.27^**^	-				
6. M-Stigma	-0.29^**^	-0.28^**^	0.60^**^	-0.36^**^	-0.39^**^	-			
7. C-Promotion	0.42^**^	0.19^**^	-0.31^**^	0.41^**^	0.23^**^	-0.30^**^	-		
8. C-Prevention	0.28^**^	0.34^**^	-0.21^**^	0.24^**^	0.35^**^	-0.19^**^	0.42^**^	-	
9. C-Stigma	-0.39^**^	-0.28^**^	0.50^**^	-0.25^**^	-0.24^**^	0.43^**^	-0.42^**^	-0.40^**^	-
Mean	20.79	16.49	21.75	20.69	17.46	21.54	20.87	16.70	22.09
Standard Deviation	3.95	3.55	8.04	3.71	3.11	8.06	3.83	3.41	8.52

Note: ^**^p<0.01; F, Father; M, Mother; C, Children

### Analysis of the intergenerational transmission effects of regulatory focus and loneliness stigma

Linear mixed model analyses revealed that fathers’ promotion focus (β = 0.29, SE = 0.04; F(465) = 44.85, p<0.001; t = 6.70, p<0.001) and mothers’ promotion focus (β = 0.30, SE = 0.05; F(465) = 42.53, p<0.001; t = 6.52, *p* < 0.001) significantly and positively predicted children’s promotion focus. Fathers’ prevention focus (β = 0.22, SE = 0.04; F(465) = 25.48, p<0.001; t = 5.05, *p* < 0.001) and mothers’ prevention focus (β = 0.28, SE = 0.05; F(465) = 30.18, p<0.001; t = 5.49, *p* < 0.001) significantly and positively predicted children’s prevention focus. In addition, the father’s loneliness stigma (β = 0.38, SE = 0.05; F(465) = 54.73, p<0.001; t = 7.40, *p* < 0.001) and mother’s loneliness stigma (β = 0.21, SE = 0.05; F(465) = 17.12, p<0.001; t = 4.14, *p* < 0.001) significantly and positively predicted children’s loneliness stigma. This suggests that regulatory focus and loneliness stigma have intergenerational transmission effects.

### An APIM analysis of the impact of parent Promotion Focus on Loneliness Stigma

The results of the APIM model analysis revealed (see Table 2) that the actor effect values of fathers’ and mothers’ promotion focus were − 0.89 (*p* < 0.001, 95% CI [-1.16, -0.63]) and − 0.74 (*p* < 0.001, 95% CI [-1.01, -0.48]), respectively, with standardized main effect values of -0.42 and − 0.35, which satisfied the requirements for conducting the prerequisites for k-value calculations. Equivalence of the actor effect values for fathers’ and mothers’ promotion focus was tested using χ^2^ analyses, and no significant difference was found (*p* = 0.50, 95% CI [-0.59, 0.29]). Additionally, the partner effect values for fathers and mothers were − 0.33 (*p* = 0.004, 95% CI [-0.57, -0.12]) and − 0.28 (*p* = 0.022, 95% CI [-0.52, -0.05]) for the standardized partner effects, respectively, with standardized partner effect values of -0.16 and − 0.13. χ^2^ analyses revealed that the restricted and unrestricted models for parental partner effects revealed no significant differences (*p* = 0.79, 95% CI [-0.34, 0.46]).

**Table 2:** Results of an APIM analysis of the effects of parental promotion focus on the loneliness stigma

Effects	Effects value	Standardized effect size	95% CI	*p*
F-intercept	21.70		[20.58, 22.85]	< 0.001
F-actor effect	-0.89	-0.42	[-1.16, -0.63]	< 0.001
F-partner effect	-0.33	-0.16	[-0.57, -0.12]	0.004
F-*k*	0.31		[0.04, 0.76]	
M-intercept	21.50		[20.30, 22.72]	< 0.001
M- actor effect	-0.74	-0.35	[-1.01, -0.48]	< 0.001
M-partner effect	-0.28	-0.13	[-0.52, -0.05]	0.022
M-*k*	0.45		[0.13, 1.07]	

Note: F, Father; M, Mother

The study further calculated the k-values where the father’s and mother’s k-values were 0.31 and 0.45 respectively. To discern the nature of pairwise patterns, confidence intervals for the k-values were estimated using the Bootstrap method. The results displayed a 95% CI for the father’s k-value ranging from 0.04 to 0.76, indicating that his pairwise pattern lies between the actor-only pattern and the coupling pattern. The 95% CI for mothers’ k-values ranged from 0.13 to 1.07, indicating that their pairwise patterns were between the coupled pattern. Additionally, the study tested the discriminability of the model by equating both the subject and object effects amongst parents and assessing the significance of changes in the model’s chi-square value. The findings revealed χ^2^ of 18.32 with 11 degrees of freedom (*p* = 0.08). Given the *p*-value was below the threshold of 0.20, the model was determined to be distinguishable.

### APIM analysis of the effect of parental prevention focus on loneliness stigma

The results demonstrated (see Table 3) that the actor effect values for prevention focus for fathers and mothers were − 0.70 (*p* < 0.001, 95% CI [-0.94, -0.46]) and − 0.87 (*p* < 0.001, 95% CI [-1.16, -0.58]), respectively, with standardized actor effect values of -0.29 and − 0.36, which satisfy the k-values for performing the prerequisites. Tests of equivalence of actor effect values for prevention focus for fathers and mothers using χ^2^ analyses showed a nonsignificant difference (*p* = 0.39, 95% CI [-0.21, 0.56]). In addition, partner effect values were − 0.35 (*p* < 0.001, 95% CI [-0.58, -0.12]) and − 0.50 (*p* < 0.001, 95% CI [-0.77, -0.22]) for fathers and mothers, respectively, with standardized partner effect values of -0.14 and − 0.20. The results of the χ^2^ analysis showed that the restriction model and the unrestricted model of the parental partner effect were not significantly different (*p* = 0.44, 95% CI [-0.52, 0.22]).

**Table 3:** Results of the APIM analysis of the impact of parental prevention focus on loneliness stigma

Effects	Effects value	Standardized effect size	95% CI	*p*
F-intercept	21.82		[20.73, 22.97]	< 0.001
F-actor effect	-0.70	-0.29	[-0.94, -0.46]	< 0.001
F-partner effect	-0.35	-0.14	[-0.58, -0.12]	0.003
F-*k*	0.71		[0.27, 1.46]	
M-intercept	21.84		[20.68, 23.03]	< 0.001
M- actor effect	-0.87	-0.36	[-1.16, -0.58]	< 0.001
M-partner effect	-0.50	-0.20	[-0.77, -0.22]	< 0.001
M-*k*	0.40		[0.12, 0.86]	

Note: F, Father; M, Mother

The study further calculated the k-values, where the k-values for fathers and mothers were 0.71 and 0.40 respectively. The 95% CI of the fathers’ k-values ranged from 0.27 to 1.46, including 1, which indicates that their pairwise pattern is a couple pattern. The 95% CI of the mothers’ k-values ranged from 0.12 to 0.86, which indicates that their pairwise pattern is between the actor-only pattern and the couple pattern. In addition, the models were tested to see if they were distinguishable models. The results showed χ^2^ (11) = 47.87, *p* < 0.001, indicating that the model is distinguishable.

### A mediation model analysis of the effect of parental promotion focus on the loneliness stigma of college children

**Fig. 4:**
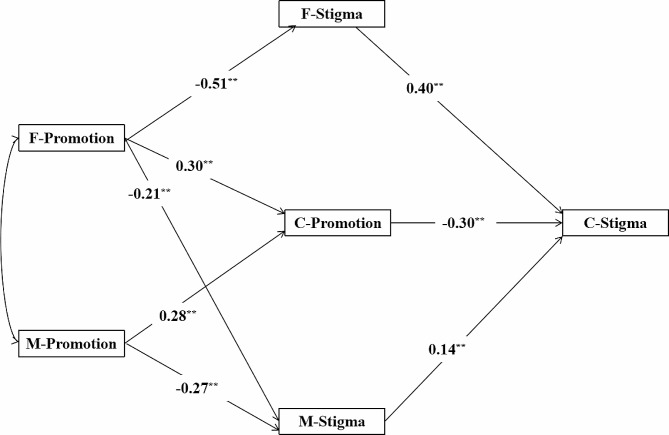
A mediation model of the effect of parental promotion focus on the loneliness stigma of college children **Note**: F, Father; M, Mother; C, Children. ^**^p<0.01

Based on the analysis of correlation, intergenerational transmission effects, and APIM, the study further constructed a mediation model of the effect of parental promotion focus on college children’s loneliness stigma. The results showed (see Fig. 4) that the predictive effect of maternal promotion focus on paternal loneliness stigma was not significant (β= -0.07, *p* = 0.120). After removing this path, the model had good fit indices: χ^2^/df = 2.852, RMSEA = 0.063, SRMR = 0.043, CFI = 0.983, TLI = 0.968, IFI = 0.983, and GFI = 0.976. Specifically, fathers’ promotion focus negatively predicted the influence of fathers’ (β= -0.51, *p* < 0.001) and mothers’ (β= -0.21, *p* < 0.001) loneliness stigma and positively predicted children’s promotion focus (β = 0.30, *p* < 0.001); mothers’ promotion focus negatively predicted mothers’ (β= -0.27, *p* < 0.001) loneliness stigma and positively predicted children’s promotion focus (β = 0.28, *p* < 0.001); loneliness stigma of fathers (β = 0.40, *p* < 0.001) and mothers (β = 0.14, *p* = 0.029) positively predicted loneliness stigma of children; and promotion focus of children negatively predicted loneliness stigma of children (β= -0.30, *p* < 0.001).

The significance of the indirect paths through which parents’ promotion focus affects college children’s loneliness stigma was tested using the Bootstrap method. The results showed (see Table 4) that fathers’ and mothers’ promotion focus could affect their children’s loneliness stigma through five indirect paths, and the bootstrap 95% CI for each path did not include 0. The value of the total indirect effect of fathers’ promotion focus on college student children’s loneliness stigma was − 0.323, and the value of the total indirect effect of mothers’ promotion focus on college student children’s loneliness stigma was − 0.122.

**Table 4:** Bootstrap analysis of the significance of the mediating path of parental promotion focus for college children

Path	Indirect Effect	Boot SE	95% CI
F-Promotion→F-Stigma→C-Stigma	-0.204	0.043	[-0.296, -0.125]
F-Promotion→C-Promotion→C-Stigma	-0.090	0.022	[-0.139, -0.051]
F-Promotion→M-Stigma→C-Stigma	-0.029	0.017	[-0.071, -0.002]
M-Promotion→C-Promotion→C-Stigma	-0.084	0.019	[-0.129, -0.056]
M-Promotion→M-Stigma→C-Stigma	-0.038	0.021	[-0.083, -0.001]

Note: F, Father; M, Mother; C, Children

### A mediation model analysis of the effect of parental prevention focus on the loneliness stigma of college children

**Fig. 5:**
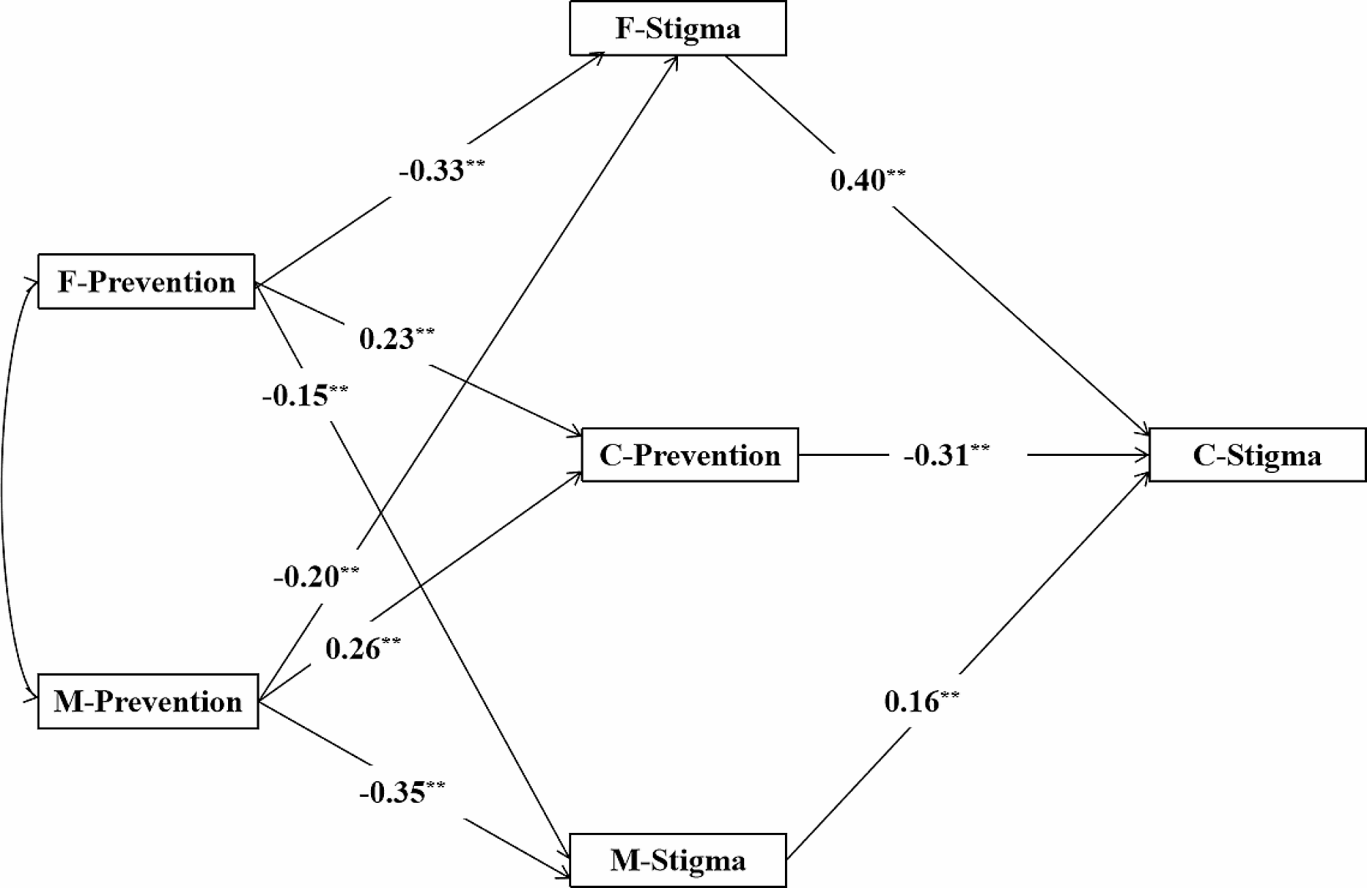
A mediation model of the effect of parental prevention focus on the loneliness stigma of college children **Note**: F, Father; M, Mother; C, Children. ^**^p<0.01

Tests of the mediation model showed (see Fig. 5) good fit indices for each model: χ2/df = 1.966, RMSEA = 0.045, SRMR = 0.020, CFI = 0.991, TLI = 0.983, IFI = 0.991, and GFI = 0.984. Specifically, fathers’ prevention focus negatively predicted the loneliness stigma of both fathers’ (β= -0.33 (*p* < 0.001) and mothers’ (β= -0.15, *p* = 0.002) loneliness stigma and positively predicted children’s prevention focus (β = 0.23, *p* < 0.001); mothers’ prevention focus negatively predicted fathers’ (β= -0.20, *p* < 0.001) and mothers’ (β= -0.35, *p* < 0.001) loneliness stigma and positively predicted children’s prevention focus (β = 0.26, *p* < 0.001); fathers’ (β = 0.40, *p* < 0.001) and mothers’ (β = 0.16, *p* = 0.008) loneliness stigmas positively predicted children’s loneliness stigma; and children’s prevention focus negatively predicted children’s loneliness stigma (β= -0.31, *p* < 0.001).

The significance of each mediating path was assessed using the Bootstrap method. The findings showed (see Table 5) that fathers’ and mothers’ prevention focus could affect college children’s loneliness stigma through six indirect paths, and the bootstrap 95% CI for each path did not include 0. The value of the total indirect effect of the father’s prevention focus on college children’s loneliness stigma was − 0.227, and the value of the total indirect effect of the mother’s prevention focus on college children’s loneliness stigma was − 0.217.

**Table 5:** Bootstrap Analysis of Parental Prevention Focus on Mediating Pathway Significance Test for College Children

Path	Indirect Effect	Boot SE	95% CI
F-Prevention→F-Stigma→C-Stigma	-0.132	0.033	[-0.220, -0.089]
F-Prevention→C-Prevention→C-Stigma	-0.071	0.020	[-0.123, -0.044]
F-Prevention→M-Stigma→C-Stigma	-0.024	0.017	[-0.074, -0.004]
M-Prevention→F-Stigma→C-Stigma	-0.080	0.034	[-0.182, -0.045]
M-Prevention→C-Prevention→C-Stigma	-0.081	0.025	[-0.156, -0.056]
M-Prevention→M-Stigma→C-Stigma	-0.056	0.032	[-0.140, -0.013]

Note: F, Father; M, Mother; C, Children

## Discussion

This study examined the relationship between regulatory focus and loneliness stigma from individual, parent-child, and couple perspectives, using Chinese families as research subjects. It was discovered that both the promotion focus and the prevention focus were negatively associated with loneliness stigma, suggesting H1 but not H2. However, individuals with differently predominant regulatory focuses adopt different means of regulation in the process of goal achievement. However, it is possible to achieve similar desired end-states and outcomes in a given situation or task [48]. In Grant et al.'s study, it was revealed that both promotion and prevention focus positively predicted individuals’ optimism, indices of well-being, and positive coping styles and negatively corrected depression and obsessive-compulsive disorder [52]. In Adams et al.'s study, individuals high in promotion focus had a higher willingness to re-establish social connections and cooperate after experiencing social exclusion, while individuals high in prevention focus also showed a higher willingness to cooperate after being perceived as safe [53]. The present study reached the consistent conclusion that two kinds of regulatory focus play a similar role in the improvement of loneliness stigma. This study contributes to the validation of the regulatory focus theory in the realm of interpersonal relationships and enhances our understanding of the impact of promotion and prevention focus on individuals’ daily lives.

In previous research, promotion focus has been identified as having a positive impact on the establishment and development of interpersonal relationships, while prevention focus is usually associated with negative outcomes [54]. However, prevention focus also plays a positive role in reducing loneliness stigma. This may be because individuals with a predominantly prevention focus are likely to take appropriate measures to mitigate the harm of stigma when they realize that the public stigma of loneliness can be detrimental to their own intergroup relationships or that the self-stigma of loneliness can affect their lives and moods. In addition, the influence of regulatory focus on information choice is similarly affected by situational factors as well as information load [55]. Yoon et al. observed that promotion-focused individuals pay attention to positive information in the presence of a high information load, while prevention-focused ones care more about positive information in the presence of a low information load [56]. Therefore, when prevention-focused individuals encounter a small amount of negative information related to loneliness, they prioritize positive cues, which helps reduce loneliness stigma. Hence, similar to promotion focus, prevention focus also contributes to the reduction of loneliness stigma.

Parental regulatory focus positively predicted children’s regulatory focus, suggesting that H3 is valid. In a study conducted by Tabuchi et al., it was observed that only prevention focus exhibited an intergenerational transfer effect, while the correlation between parent-child promotion focus was not significant [57]. In the present study, both promotion focus and prevention focus were observed to have intergenerational transfer effects among middle-aged parents and college-aged children. The study by Tabuchi et al. used elderly parents and middle-aged children as survey respondents. The age difference of the subjects may be the reason for the inconsistent results of the study. The college-aged children were not completely separated from their original families and were significantly more likely than middle-aged adults to receive family education as well as the frequency of contact with their parents and the amount of time they spent living together [58]. This results in the influence of middle-aged parents on college children being higher than the influence of older parents on middle-aged children. In addition, Higgin and Silberman argued that different regulatory-focused parents adopt either nurturance-oriented parenting or security-oriented parenting, which leads children and young adults to develop regulatory focus tendencies similar to those of their parents [59]. The findings of the present study align with this argument. It suggests that prevention-focused tendencies established during childhood tend to remain relatively stable throughout various life developmental stages, whereas promotion-focused tendencies may exhibit inconsistencies between childhood and young adulthood, particularly after reaching middle age.

The study revealed that parental loneliness stigma is positively correlated with children’s loneliness stigma, indicating that H4 is valid. This result suggests that individuals’ loneliness stigma is to some extent influenced by parental loneliness stigma. In the process of raising and educating their children, parents transmit both their own advantages and disadvantages to their children and influence their psychological and behavioral development [60]. The effects of parenting on offspring loneliness and stigma have been explored in previous studies [35, 61]. However, the literature has not addressed the phenomenon of intergenerational transmission of loneliness stigma. The present study is the first to analyze this phenomenon, reflecting certain innovative insights. In addition, previous research on the intergenerational transmission effect of loneliness has identified that mothers are more predictive of adult children than fathers [35]. In contrast, the present study reached inconsistent conclusions in the area of loneliness stigma. Unlike loneliness, loneliness stigma, as a negative social evaluation, responds to an individual’s attitude toward loneliness and the negative effects it triggers. Evidence points to the more important role of fathers in the attitudinal and social cognitive development of their children [62, 63]. Therefore, the effect of fathers’ loneliness stigma on children’s loneliness stigma is more prominent than that of mothers.

In the analysis of the APIM model, it was found that parental regulatory focus had a negative impact on both their own loneliness stigma and their spouse’s loneliness stigma, confirming H5. The establishment of the actor effect reaffirms H1 and H2. The presence of the partner effect supports the that the elements within the family system are interdependent [46]. The presence of partner effects in couples has been found in previous studies on the APIM of loneliness and interpersonal stigma [44, 64]. The present study further suggests that the couple as a whole and the regulatory focus of either partner can help to reduce the loneliness stigma of the spouse. Furthermore, it was pointed out that there is a gender asymmetry in the couple’s partner effect [65]. The present study, however, did not reach similar conclusions. This may be because loneliness stigma is not a relatively stable trait, and changes with the influence of the external environment, culture, media, individual experiences, and significant others’ perceptions [66]. Couples are a tightly knit whole, and the perceptions and behaviors of either partner may affect the loneliness stigma of the spouse.

The study developed a mediation model based on an analysis of the relationship between regulatory focus and loneliness stigma, intergenerational transmission effects, and the APIM. The findings revealed that parental loneliness stigma and children’s regulatory focus play a mediating role in the effect of parental regulatory focus on children’s loneliness stigma. It was shown that H6, H7, and H8 were valid. The findings suggest that parents’ regulatory focus can influence the interaction patterns between parents and children, which in turn influences children’s regulatory focus and has a profound effect on their future loneliness stigma. Moreover, parents’ regulatory focus can affect their own and their spouse’s loneliness stigma, and parents’ attitudes and evaluations of loneliness, in turn, can affect their children’s loneliness stigma. The results of this study contribute to a better understanding of the mechanisms by which parental regulatory focus influences children’s loneliness stigma and may also inform the development of intervention practices.

This research holds practical value, offering insights for the enhancement of psychological counseling and mental health education. This study found that both promotion and prevention regulatory focus can play a role in reducing loneliness stigma, a threat to individual’s mental health. However, depending on a particular context or task, a certain regulatory focus may prove more advantageous than the other [67]. This suggests that tailored interventions should be developed to address the stigma of loneliness in individuals with differing regulatory focuses. In addition, regulatory focus can be influenced by self-regulation experience, which manifests itself as a chronic and stable personality trait, or manifests as a temporary motivation according to current situations or tasks [20]. Therefore, when intervening in the stigma of loneliness, the practitioners can achieve the goal of reducing the stigma of loneliness by activating specific temporary regulatory focus motivation based on the situation.

The results of this study on the APIM model analysis and intergenerational transmission effect of regulatory focus and loneliness stigma showed that the regulatory focus and loneliness stigma of parents and spouses can affect individual loneliness stigma. This suggests that there is a mutual influence among family members, and that a member’s change can affect the psychology and behavior of the others. Therefore, when intervening in the individual stigma of loneliness, the practitioners should consider the influence of family factors and the attitudes of parents and spouses towards loneliness. In addition, parents should realize that the lasting impact of their regulatory focus on children’s psychology and behavior. Therefore, parents should strive to align their parenting style more closely with their child’s psychological needs. The results indicate that interventions targeting behavioral and cognitive patterns within parent-child or spousal relationships could concurrently be effective in reducing loneliness stigma within the context of marriage and family therapy.

### Limitations

First, the study adopted convenience sampling and recruited subjects from a single university, potentially compromising the representativeness of the sample. Moreover, the study only investigated college students and their parents, raising uncertainties about the applicability of the results to different age demographics. As suggested by previous study, the stigma of loneliness in college students presents different characteristics from other adult age groups [37]. Therefore, it should be cautious in concluding the findings in other age groups, educational levels, marital statuses, and regions.

Second, the present study struggles to establish causality between variables and mitigate the influence of the social desirability bias. To rectify these issues, the future studies can consider adopting experimental and longitudinal research, combining multiple approaches such as vignette task and Implicit Association Test, to deal with the limitations in current study. Meanwhile, the study only explored the parental effect on children, while the filial generation can also affect their parents as depicted in family systems theory. Therefore, the experimental and longitudinal research facilitates further understanding of the mutual effect of regulatory focus and stigma of loneliness among family members.

Third, the current study’s context was confined to Chinese society, raising questions regarding the generalizability of the findings across different cultural settings. Individuals in different cultures do not differ in their need for interpersonal relationships and their level of reliance on social support, which may affect their attitudes toward loneliness. For example, individualistic cultures show higher levels of acceptance and lower levels of loneliness stigma compared to collectivistic cultures [68]. Additionally, regulatory focus shows some cultural differences [69]. Therefore, future research is necessitated to examine whether these findings hold true in a variety of cultural environments.

Fourth, the present study did not explore the role of individuals’ personalities and other psychological traits in the effect of regulatory focus on loneliness stigma. Previous research has noted that although both promotion and prevention focus are positively associated with well-being, they do not have the same mediating mechanisms [52]. Moreover, parent-child relationship and parenting styles exert great impact on the intergenerational transmission of regulatory focus and loneliness stigma. Therefore, a comprehensive analysis of family dynamics is warranted to further understand how parental regulatory focus influences their children’s loneliness stigma.

## Conclusion

The present study revealed that regulatory focus is inversely related to loneliness stigma, with both demonstrating patterns of intergenerational transmission. In addition, further analyses of the APIM and the mediation model indicated that parental loneliness stigma and children’s regulatory focus mediated the effects of parental regulatory focus on children’s loneliness stigma. This finding suggests that appropriate regulatory focus can be activated in different contexts to better reduce loneliness stigma. In addition, these findings highlight the opportunity to reduce individual loneliness stigma from a familial approach, by targeting interventions at the regulatory focus and loneliness stigma of spouses or parents.

## Data Availability

The datasets used or analyzed during the current study are available from the corresponding author upon reasonable request.

## Abbreviations

RFQ: Regulatory Focus Questionnaire
SLS: Stigma of Loneliness Scale
APIM: Actor-Partner Interdependence Model
SSL: Self-Stigma of Loneliness
PSL: Public Stigma of Loneliness
LME: Linear mixed model
CI: Confidence interval
RMSEA: Root-mean-square error of approximation
SRMR: Speech-to-reverberation modulation energy ratio
CFI: Comparative fit index
IFI: Incremental fit index
GFI: Goodness-of-fit index
TLI: Tucker-Lewis index
